# Polymorphisms in Toll-Like Receptors 2, 4, and 9 Are Highly Associated with Hearing Loss in Survivors of Bacterial Meningitis

**DOI:** 10.1371/journal.pone.0035837

**Published:** 2012-05-25

**Authors:** Gijs Th J. van Well, Marieke S. Sanders, Sander Ouburg, A. Marceline van Furth, Servaas A. Morré

**Affiliations:** 1 Department of Pediatric Infectious Diseases, Immunology and Rheumatology, VU University Medical Center, Amsterdam, The Netherlands; 2 Laboratory of Immunogenetics, Department of Medical Microbiology, VU University Medical Center, Amsterdam, The Netherlands; 3 Department of Pediatrics, Maastricht University Medical Center, Maastricht, The Netherlands; 4 Department of Surgery, Antonius Hospital, Nieuwegein, The Netherlands; 5 Institute of Public Health Genomics, Department of Genetics and Cell Biology, Research Institutes CAPHRI (School for Public Health and Primary Care) and GROW (School for Oncology & Developmental Biology), Faculty of Health, Medicine & Life Sciences, University of Maastricht, Maastricht, The Netherlands; Oxford University, VietNam

## Abstract

Genetic variation in innate immune response genes contributes to inter-individual differences in disease manifestation and degree of complications upon infection. We recently described an association of single nucleotide polymorphisms (SNPs) in *TLR9* with susceptibility to meningococcal meningitis (MM). In this study, we investigate the association of SNPs in multiple pathogen recognition and immune response genes with clinical features that determine severity and outcome (especially hearing loss) of childhood MM and pneumococcal meningitis (PM). Eleven SNPs in seven genes (*TLR2*, *TLR4, TLR9*, *NOD1*, *NOD2*, *CASP1,* and *TRAIL*) were genotyped in 393 survivors of childhood bacterial meningitis (BM) (327 MM patients and 66 PM patients). Genotype distributions of single SNPs and combination of SNPs were compared between thirteen clinical characteristics associated with severity of BM. After correction for multiple testing, *TLR4*+896 mutant alleles were highly associated with post-meningitis hearing loss, especially MM (*p*  = 0.001, OR 4.0 for BM, *p*  = 0.0004, OR 6.2 for MM). In a multigene analysis, combined carriership of the *TLR2*+2477 wild type (WT) with *TLR4*+896 mutant alleles increases the risk of hearing loss (*p*<0.0001, OR 5.7 in BM and *p*  = 0.0001, OR 7.6 in MM). Carriage of one or both mutant alleles in *TLR4*+896 and *TLR9* -1237 increases the risk for hearing loss (*p*  = 0.0006, OR 4.1 in BM). SNPs in immune response genes contribute to differences in clinical severity and outcome of BM. The TLR system seems to play an important role in the immune response to BM and subsequent neuronal damage as well as in cochlear inflammation. Genetic markers may be used for identification of high-risk patients by creating prediction rules for post-meningitis hearing loss and other sequelae, and provide more insight in the complex immune response in the CNS possibly resulting in new therapeutic interventions.

## Introduction

Bacterial meningitis (BM) is a serious infectious disease of the central nervous system (CNS). Despite worldwide immunization programs and improvement of antimicrobial and anti-inflammatory therapy, BM is still responsible for substantial mortality in both developing and developed countries. The clinical presentation, severity and outcome of BM are highly diverse. Mortality is four to ten percent [1] and 20% of survivors develop neurological sequelae, ranging from learning and behavioral disorders to deafness, seizures, and motor deficits in 13% of cases [2]. Considerable evidence implicates that genetic variation in microbial recognition genes is associated with altered host responses to infection and the degree of post-infectious complications [3].

Pathogen recognition receptors (PRRs), present on various cells, including microglia and astrocytes inside the CNS [4], recognize pathogen-associated molecular patterns (PAMPs). High affinity binding activates nuclear factor kappa B (NFκB) and the subsequent genetic transcription of pro-inflammatory cytokines. Toll-like receptors (TLRs) and nucleotide-binding oligomerisation domain-containing proteins (NODs) are two major groups of PRRs.

We recently reported an association of single nucleotide polymorphisms (SNPs) in *TLR9* with susceptibility to meningococcal meningitis (MM) [5]. Several studies have shown that SNPs in innate immune response genes affect the clinical course of both meningococcal and pneumococcal infections [6]. Three papers focused on genetic variation in innate immune response genes and BM specifically [5], [7], [8].

This study aims to assess associations of single SNPs as well as combinations of SNPs with severity and clinical outcome in post-meningitis children. The distribution of eleven SNPs in seven genes involved in pathogen recognition were related to thirteen clinical or laboratory variables of BM severity as described in literature.

We studied TLR2, an extracellular receptor that recognizes lipoteichoic acid (LTA), present in the cell wall of *Streptococcus pneumoniae* [9], and meningococcal porin [10]. TLR2 activation triggers intracellular signaling via myeloid differentiation protein 88 (MyD88), resulting in pro-inflammatory cytokine production. *TLR2*−/− mice, intracerebrally infected with pneumococci, showed higher mortality, aggravated brain bacterial loads, higher tumor necrosis factor alpha (TNF-α) concentrations in brain homogenates, and more damage to the blood-brain barrier (BBB) [11]. *TLR2* -16934 SNPs are associated with higher risk of sepsis caused by Gram-positive bacteria [12]. *TLR2*+2477 mutants have been associated with a reduced responsiveness to *Staphylococcus aureus* infections [13].

TLR4 recognizes lipopolysacharide (LPS) in the outer membrane of *Neisseria meningitidis.* TLR4 triggering activates intracellular signaling via MyD88, resulting in NFkB transcription and subsequent cytokine production. *TLR4*+896 mutants cause hypo-responsiveness to LPS [14] and enhance susceptibility to invasive meningococcal [15]–[17] and pneumococcal infections [18]. *TLR4*+896 mutants are also associated with an increased risk of developing invasive disease in patients with pneumococcal infections [18].

TLR9 is an intracellular PRR which recognizes unmethylated Cytosine-phosphate-Guanine (CpG) motives in bacterial DNA [19]. Meningococcal [5] and pneumococcal [20] CpG DNA enters the cell by endocytosis and binds to TLR9. TLR9 activation triggers the MyD88 dependant pro-inflammatory pathway. We recently demonstrated that the *TLR9*+2848 SNP is associated with decreased susceptibility to MM [5].

NODs are intracellular pattern recognition receptors containing a Caspase-recruitment domain (CARD). NOD1 and NOD2 recognize degradation products of peptidoglycan (PGN) [21], a unique and essential component of the cell wall of the vast majority of bacteria. *NOD1*−/− mice are more susceptible than wild type (WT) mice to early sepsis after intranasal administration of pneumococci [22]. Isolated cultures of murine astrocytes and microglia constitutively express robust levels of NOD2 after exposure to both *N. meningitidis* [23] and *S. pneumoniae* [24]. *NOD1*+32556 and *NOD2+2209*, *NOD2*+2722, and *NOD2*+3020 SNPs are associated with inflammatory bowel disease [25], [26] and share a signaling defect in response to LPS and PGN [27].

Caspases play an essential role in apoptosis. Activation of intracellular caspase-1 (CASP1) upon pathogen recognition by TLRs and NODs defends the host against infection by secretion of the pro-inflammatory cytokines IL-1β and IL-18 via the IL-1 receptor, and by the induction of apoptosis of infected cells inside the cell in a molecular structure called the inflammasome [28]. CASP1 levels are up regulated in CSF of patients with BM and correlate with clinical outcome, assessed by the Glasgow Coma Scale. *CASP1*−/− mice intracerebrally infected with *S. pneumoniae* show a significantly attenuated increase of IL-1β, lower CSF leukocytes and an improved clinical status [29]. *CASP1* haplotypes are associated with decreased serum IL-18 levels [30].

TNF-related apoptosis-inducing ligand (TRAIL) is a protein that induces caspase driven apoptosis upon activation. TRAIL limits granulocyte driven inflammation in BM. TRAIL levels are elevated in CSF of patients with BM. *TRAIL* −/− mice show prolonged inflammation, augmented clinical impairment, and increased apoptosis in the hippocampus following intrathecal application of pneumococcal cell wall solution [31]. A highly polymorphic region in *TRAIL,* including the *TRAIL* -692 SNP, has been identified but not yet linked to human disease [32].

This study aims to describe significant associations of SNPs in innate immune response genes with severity and outcome in survivors of childhood BM by comparing genotype distributions between thirteen clinical severity parameters, including hearing loss.

## Results

*Table 1* shows the patient characteristics and the distribution of the severity variables in our study population. Thirteen clinical severity variables were studied, including duration of clinical illness before admission, rectal temperature at admission, and the laboratory variables CSF leukocyte number, CSF/blood glucose ratio, CSF protein concentration, blood leukocyte number, C-reactive protein (CRP) concentrations, blood culture (positive for meningitis causing organism), convulsions, disturbed consciousness at admission, ICU-admission, hearing loss, and clinical diagnosis at discharge (meningitis with or without sepsis). Different numbers within groups are due to missing or non-determined data in patient records.

**Table 1:** Patient characteristics and distribution of 13 clinical severity variables in 393 children with bacterial meningitis.

Continuous variables	Median (range) total BM group		Median MM Group		Median PM group		Total BM (MM/PM)
							*n*
Clinical characteristic/Severity variable							
Age at admission (years)	2,2 (0–9,5)		2.5		1.0		393 (327/66)
Duration clinical illness before admission (days)	1,0 (0,5–11,0)		1.0		2.0		387 (321/66)
Rectal temperature (°C)	39,4 (35,0–41,8)		39.2		39.8		362 (299/63)
CSF leukocytes (n/µL)	1013 (0–120810)		1243		600		353 (297/56)
CSF blood/glucose ratio	0,30 (0–1,77)		0.29		0.18		278 (236/42)
CSF protein concentration (g/l)	1,4 (0,01–9,33)		1.7		2.1		324 (58/266)
Blood leukocytes (nx10^∧^9/l)	16,8 (1,3–93,5)		16.7		20.3		384 (319/65)
CRP concentration (mg/L)	137 (0–768)		135		158		234 (190/44)
**Dichotomous variables**	**Total BM Group**	**%**	**MM**	**%**	**PM N**	**%**	**Total determined**
	**n**		**n**		**n**		**n**
Clinical characteristic/Severity variable							
Male gender	219	56	174	53	45	68	393 (327/66)
Blood culture positive	188	55	143	44	45	68	345 (286/59)
Convulsions	52	13	30	9	22	33	393 (327/66)
Consciousness disturbed	261	69	215	66	46	70	379 (316/63)
ICU-admission	77	20	69	21	8	12	392 (326/66)
Hearing loss	27	7	13	4	14	21	393 (327/66)
Meningitis with sepsis	170	43	147	45	23	35	393 (327/66)

Different numbers within groups are due to missing data in patient records.

Abbreviations: BM: bacterial meningitis, MM: meningococcal meningitis, PM: pneumococcal meningitis, CSF: cerebrospinal fluid, CRP: C-reactive protein, ICU: intensive care unit.

*Table 2* shows the genotype distributions of eleven studied SNPs in our study population, for MM and PM patients. No significant differences in genotype distributions were observed between MM and PM patients.

**Table 2:** Genotypes of 11 studied polymorphisms in patients with meningococcal and pneumococcal meningitis.

SNP	Genotype	Total BM	%	MM	%	PM	%
		(*n* = 393)		(*n* = 327)		(*n* = 66)	
*TLR2* -16934 T>A	TT	107	27	89	27	18	27
(rs4696480)	TA	184	47	150	46	34	52
	AA	102	26	88	27	14	21
*TLR2*+2477 G>A	GG	357	91	298	91	59	89
(rs5743708)	GA	34	9	27	8	7	11
	AA	2	1	2	1	0	0
*TLR4*+896 A>G	AA	343	87	283	87	60	91
(rs4986790)	AG	37	9	33	10	4	6
	GG	13	3	11	3	2	3
*TLR9* -1237 T>C	TT	291	74	244	75	47	71
(rs5743836)	TC	95	24	76	23	18	29
	CC	7	2	7	2	0	0
*TLR9*+2848 G>A	GG	90	23	79	24	11	17
(rs352140)	GA	193	49	162	50	31	47
	AA	110	28	86	26	24	36
*NOD1*+32556 T- > GG	T-T-	225	57	183	56	42	64
(rs6958571)	T-GG	148	38	126	39	22	33
	GGGG	20	5	18	6	2	3
*NOD2*+2209 C>T	CC	349	89	290	89	59	89
(rs2066844)	CT	37	9	30	9	7	11
	TT	7	2	7	2	0	0
*NOD2*+2722 G>C	GG	384	98	318	97	66	100
(rs2066845)	GC	7	2	7	2	0	0
	CC	2	1	2	1	0	0
*NOD2*+3020 ins C	−/−	380	97	316	97	64	97
(rs5743293)	−/C	12	3	10	3	2	3
	C/C	1	0	1	0	0	0
*CASP1*+8404 A>G	AA	234	60	195	60	39	59
(rs2282659)	AG	132	34	112	34	20	30
	GG	27	7	20	6	7	11
*TRAIL* -692 T>C	TT	317	81	266	81	51	77
(rs365238)	TC	69	18	56	17	13	20
	CC	7	2	5	2	2	3

Abbreviations: SNP: single nucleotide polymorphism, BM: bacterial meningitis, MM: meningococcal meningitis, PM: pneumococcal meningitis, TLR: Toll-like receptor, NOD: nucleotide oligomerization domain protein, CASP: caspase, TRAIL: Tumor necrosis factor-related apoptosis inducing ligand.

### Power Analyses

A priori power analyses show that we have sufficient power at the 80% threshold in BM patients. However for the rare SNPs we were not able to reach the 80% threshold in the PM and MM subgroups. Since BM is relatively rare it is hard to obtain large numbers of clinically well defined patients.

### Genotype and Allele Frequencies

Continuous variables were compared by T-tests or Mann-Whitney U tests. Duration of clinical illness before admission was shorter in *TLR2 -*16934-AA carriers compared to TT and TA carriers (mean 1.3±1.4 days in AA carriers versus 1.8±0.1 days in TT/TA carriers; *p*  = 0.0042). The other continuous variables did not show differences in genotype distributions (data not shown).

*Table 3* shows genotype distributions divided by dichotomous severity variables with a *p*-value <0.05 for MM and PM patients separately and combined. *TLR4*+896 AG or GG alleles were associated with a significantly increased risk of hearing loss (*p*  = 0.001, OR 4.0, 95% CI 1.7–9.4) especially in MM patients (*p*  = 0.0004, OR 6.2 95% CI 2.0–19.5), but was not significant for PM patients. Carriage of *TLR9* -1237 TC or CC mutant alleles was associated with an increased risk for hearing loss in BM, especially in PM patients (*p * = 0.023, OR 2,5, 95% CI 1.1-5.4 and *p*  = 0.017, OR 5.0, 95%CI 1.4–17.4, respectively). *CASP1*+8404 GG alleles were associated with a decreased incidence of fever in BM and MM patients (*p*  = 0.01, OR 0.3, 95% CI 0.1–0.8 and *p*  = 0.018 OR 0.3, 95% CI 0.1 – 0.9). These associations showed a trend, but appeared not statistically significant after stringent correction for multiple testing according to Holm-Bonferroni (*p*<0.003). No differences in genotype distributions were observed for other SNPs classified by severity variables.

**Table 3:** Genotype distributions compared between clinical severity groups.

SNP	Severity variable *n* (%)												X^2^/Fisher exact
													*p*, OR (95% CI)
	BM		MM		PM		BM		MM		PM		
	*n*	%	*n*	%	*n*	%	*n*	%	*n*	%	*n*	%	
***TLR4*** **+896**	Hearing loss						No hearing loss						AA vs AG/GG
AA	18	67	7	54	11	79	325	89	276	88	49	94	BM 0.001**, 4.0 (1.7–9,4)
AG	8	30	5	39	3	21	29	8	28	9	1	2	MM 0.0004**, 6.2 (2.0–19.5)
GG	1	4	1	8	0	0	12	3	10	3	2	4	PM NS
***TLR9*** **-1237**	Hearing loss						No hearing loss						TT vs TC/CC
TT	15	56	9	69	6	43	276	75	235	75	41	79	BM 0.023*, 2.5 (1.1–5.4)
TC	12	44	4	31	8	57	83	23	72	23	11	21	MM NS
CC	0	0	0	0	0	0	7	2	7	2	0	0	PM 0.017, 5.0 (1.4–17.4)
***CASP1*** **+8404**	Fever						No fever						AA/AG vs GG
AA	194	61	158	62	36	59	21	47	20	47	1	50	BM 0.01*, 0.3 (0.1–0.8)
AG	106	33	86	34	20	33	17	38	17	40	0	0	MM 0.018, 0.3 (0.1–0.9)
GG	17	5	12	5	5	8	7	16	6	14	1	50	PM NS

* *p*<0.05.

** *p*<0.003 (*p* significance corrected for multiple testing according to Holm-Bonferroni).

Different numbers between groups are due to missing data.

Abbreviations: SNP: single nucleotide polymorphism, OR: Odds ratio, CI: confidence interval, TLR: Toll-like receptor, BM: bacterial meningitis, MM: meningococcal meningitis, PM: pneumococcal meningitis, CASP1: caspase-1, NS: not significant.

### Multigene Analysis

Combined carriership of *TLR2*+2477 GG WT and *TLR4*+896 AG mutant alleles significantly increases the risk to develop hearing loss in BM (*p*<0.0001, OR 5.7, 95% CI 2.3–14.4, in MM patients: *p*  = 0.0001, OR 7.6, 95% CI 2.3–24, in PM patients: *p*  = 0.03, OR 13.9, 95% CI 1.3–147). Carriage of one or more mutant alleles in combined carriership of *TLR4*+896 and *TLR9* -1237 increases the risk for hearing loss in BM patients (*p*  = 0.0006, OR 4.1, 95% CI 1.8–9.4, in MM patients: *p*  = 0.02, OR 4.3 95% CI 1.3–14.2, in PM patients: *p*  = 0.003, OR 6.0, 95% CI 1.7–21.3). No significant results were observed for combined carriership of *TLR2* -16934 and *TLR2*+2477, neither for multiple SNPs in all three *NOD2* SNPs, with regard to the other severity variables (data not shown).

### Multiple Regression Analyses

Backward stepwise logistic regression with hearing loss as dependent variable resulted in a model containing blood culture, *TLR2*, *TLR4*, *TLR9*, *CASP1,* age at diagnosis, rectal temperature, CSF glucose levels, and gender. All other variables were excluded from the model during analyses. These variables accurately (97,8%) predicted BM patients with hearing loss (*p  = *0.017). Analyses were repeated for MM and PM patients separately. The same set of variables predicts hearing loss (98,2%) in PM patients *(p  = *0.043), but not in MM patients.

## Discussion

In this study we demonstrate for the first time that polymorphisms in pathogen recognition receptor genes affect the clinical course of BM. After correction for multiple testing, we conclude that duration of clinical illness before admission was shorter in *TLR2*- 16934-AA carriers compared to TT or TA carriers. *TLR4*+896 mutants are highly associated with post-meningitis hearing loss and combined carriership of the *TLR2*+2477 WT with *TLR4*+896 mutant alleles as well as the combination of *TLR4*+896 mutant alleles with *TLR9* -1237 mutant alleles significantly increases this risk. In multivariable regression analysis, the same genes are identified to predict hearing loss. In order to explain these findings, we focus on the pathogenesis of post-meningitis hearing loss. Since hearing loss occurs in more than 11% of PM survivors and 5% of MM survivors [2] most studies focus on PM. Bacteria spread from the infected subarachnoid space to the inner ear through the cochlear aqueduct, along the eighth cranial nerve or the blood vessels of the blood-labyrinth barrier [33]. In the peri-lymphatic spaces they induce a suppurative labyrinthitis. As a result, the blood-labyrinth barrier and hair cells are damaged and neurons in the spiral ganglion show apoptosis. The inner ear is, similar to the brain, a so-called immune privileged site: the number of immune competent cells is small and opsonizing agents are virtually absent under normal conditions [34]. Once bacteria have reached the inner ear, they multiply uncontrolled and bacterial autolysis occurs, leading to the release of bacterial components. The host innate immune response will be induced by recognition of these components by PRRs and lead to the transcription of cytokines by resident immunocompetent cells, the local endothelial cells and fibrocytes [34]. There is increasing evidence for a role of TLRs in mediating cochlear damage in meningitis [33]. Pneumolysin, recognized by TLR4, has been identified as a mediator of cochlear damage by its direct cytotoxic effects and via activation of the immune response [35]. Experimental meningitis due to pneumolysin-deficient pneumococci causes less hearing loss than meningitis due to WT pneumococci [36]. The innate immune response of *TLR2/TLR4* double-knockout mice to intracisternal pneumococcal infection was more severely impaired than that of mice lacking only *TLR2* or *TLR4* [37]. Inside the cochlea these receptors are also present. Activation of these local TLRs then activates MyD88, which in turn activates NFκB. *MyD88*−/− mice with experimental PM show less cochlear inflammation than infected WT mice [38]. All together, these findings show that an intact pathogen recognition system via MyD88 is important for bacterial clearance from the CNS but also responsible for inflammatory damage. TLR2, 4 and 9 are all involved in this pathway, explaining the significance of our trait analysis.

No experimental studies on the mechanisms of hearing loss in MM are published. However, since TLR2, 4 and 9 are also activated by meningococcal PAMPs [10], [21] we suggest that the mechanism of TLR induced MyD88-dependant cochlear damage might be similar to those in PM. Hyporesponsiveness to LPS leading to higher bacterial loads in *TLR4* SNP carriers might enhance MyD88-dependant inflammation and subsequent damage [14]. Upregulation of detrimental TLR4-mediated immune responses might be an alternative explanation for hearing loss in MM [39]. The *TLR4*+896 polymorphism is shown to change the ligand-binding site of the receptor and results in a LPS hypo-responsive phenotype [14]. In gene association studies, increased susceptibility to Gram-negative infections has been demonstrated [15], [16], but results from meningococcal infections did not consistently show this effect [7], [40]. We suspect that other SNPs might compensate for the TLR4 mediated hyporesponsiveness to LPS, as we show in the carrier trait analysis. Combined carriership of *TLR2*+2477 GG (WT) and *TLR4+*896**AG (mutants) increased the risk to develop post-meningitis hearing loss, indicating that *TLR2* SNPs compensate for *TLR4* mutant hyporesponsiveness. Carriage of one or more mutants in combined carriership of *TLR4*+896 and *TLR9* -1237 increases the risk for hearing loss in BM patients. The link between TLR4 and TLR9 was also shown in a murine in vitro study on macrophage cytokine production upon TLR9 mediated CpG DNA stimulation. Pretreatment of these macrophages with TLR4 mediated LPS heightened the TNF-α and IL-6 production [41]. Recent evidence indicates that the *TLR9* -1237 C allele results in an increased TLR9 initiated immune response via an extra putative binding site for NFκB [41], which may lead to increased immune-mediated cochlear damage. Another study on *TLR9* -1237 SNPs showed higher serum Interferon gamma levels in children with cerebral malaria, indicating that enhanced TLR9 mediated immune responses are also relevant inside the CNS [42].

This study consists of a unique large cohort of survivors of BM in childhood including genetic as well as clinical data. Since we used two independent cohorts, internal validation has been applied. However, interpretation of the results of this study has some limitations. Sample sizes were small for the pneumococcus- and for the hearing loss- groups. Different results were found for *S. pneumoniae* and *N. meningitidis*. This may either be due to an increased incidence of hearing loss in the PM group (21%) compared to 4% in the MM group, or to differences in pathogenesis and recognition of these two different pathogens by TLR9. TLR9 is shown to recognize both meningococci and pneumococci, but the ability to activate TLR9 was severely attenuated when bacteria had been heat-inactivated prior to stimulation *in vitro* [43]. *Since S. pneumoniae* is associated with higher bacterial loads in the CSF compared to *N. meningitidis*, the presence of a higher bacterial load in PM in the cochlea may also explain the stronger association in PM with hearing loss.

In order to assess the influence of clinical as well as genetic factors on the course of disease, larger numbers of patients and SNPs are needed preferably with prospective data and an association study should be repeated in a third, large preferable ethnically different cohort in the future to confirm the associations we found. Other SNPs involved in the bacterial infection might be relevant in BM *e.g.* macrophage migration inhibitory factor (*MIF)* Toll-interleukin 1 receptor domain containing adaptor protein (*TIRAP)* and complement mediated inflammation.

Our study provides evidence that SNPs in pathogen recognition receptor genes affect the severity of disease in a specific way. It should be mentioned that this type of study is hypothesis generating, but does not prove causation. The exact mechanisms how these SNPs affect the course of disease and hearing loss in particular are an interesting focus of further research in experimental models.

Determination of SNPs in an early stage of BM may be useful to identify children at risk to develop sequelae and to add host genetic markers to current clinical prediction rules on complications after BM such as hearing loss and academic and behavioral limitations [44], [45].

Indentifying high risk patients may also provide opportunities for specific individual therapeutic modifications to prevent the damage that occurs during the necessary immune response aimed at bacterial clearance from the CNS. Unraveling the molecular basis of the inflammatory responses inside the CNS might facilitate the development of specific and targeted anti-inflammatory therapy in high-risk patients.

## Materials and Methods

### Patients

**Figure 1:**
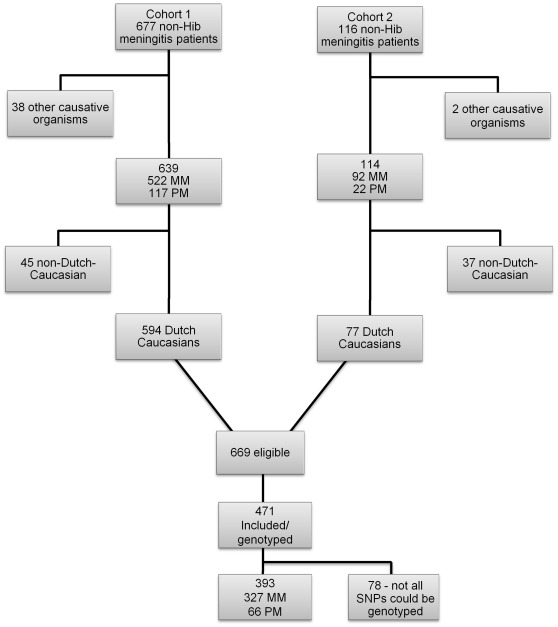
Flow chart of patient inclusion in this study.

*Figure 1* shows a flow chart of patient inclusion in this study. The cohort of school age children in part with post-meningitis hearing loss is composed of two independent cohorts. Both cohorts were described in detail in other studies [44], [45]. Both cohorts appeared to be rather similar [46] and no significant differences in genotype distributions were observed between both cohorts. Only Dutch Caucasian survivors of PM and MM were selected for this study.

Six hundred sixty nine (669) patients were eligible for our study and were asked to participate in the study and to return a sterile swab after collecting their buccal DNA. Of these patients a total of 471 (70%) returned a buccal swab and an informed consent form. Only samples with high quality DNA in which all eleven SNPs could be genotyped after three distinct Taqman and/or Lightcyler analyses were included in this study (*n*  = 393 including 327 MM patients and 66 PM patients). The median age of all patients at the time of infection was 2.2 years-old (range 0.1–9.5), 56% were male and 44% were female. All patients were selected from data on bacterial CSF isolates of the Netherlands Reference Laboratory for Bacterial Meningitis (NRLBM). The NRLBM receives approximately 90% of the isolates of Dutch meningitis patients. The diagnosis meningitis was based on the demonstration of pathogens or antigens against PM or MM in the CSF by culture. Children with ‘complex onset’ of meningitis (defined as meningitis secondary to immune deficiency states, cranial trauma, CNS surgery, and CSF shunt infections, meningitis in the neonatal period) or relapsing meningitis were not included. The Medical Ethical Committee of the VU University Medical Center approved this study.

### Clinical Variables

Data on medical history, physical examination, clinical course during hospitalization, and laboratory results were gathered retrospectively from the medical records of all patients. Detailed information on CSF culture results was collected from the NRLBM. We used clinical factors with regard to severity and outcome of BM in children that could potentially be linked to genetic variation as currently accepted in literature. These variables were in concordance with a recent systematic review summarizing the evidence regarding prognostic factors predicting death or sequelae after BM in children [47]. Only variables with a reasonable incidence (>5% cases) were included. Continuous clinical severity variables include: duration of clinical illness before admission and rectal temperature at admission. Rectal temperature was dichotomized to the presence of fever, *i.e.* rectal temperature ≥38°C. Continuous laboratory values at admission include: CSF leukocyte number, CSF/blood glucose ratio, CSF protein concentration, blood leukocyte number, CRP concentration. The dichotomous severity variables include: causing pathogen in CSF culture, meningitis causing pathogen in blood culture, convulsions present, disturbed consciousness at admission, ICU-admission, post-meningitis hearing loss, and meningitis with or without sepsis.

Convulsions were defined as convulsions reported before or at admission or during hospitalization. Hearing loss was defined as an unilateral or bilateral perceptive hearing loss >25 dB that was not present before meningitis occurred and was based on information from medical records and parental information provided from questionnaires about the children’s health [44]. Conductive hearing loss was not included.

### Missing Data

Patients in which not all 11 SNPs could be genotyped after 3 PCR assays were excluded in order to increase the continuity of our data (*figure 1*). Missing clinical data (*Table 1*) range from 0% in most cases to 40% in CRP.

### DNA Isolation

DNA was isolated from the buccal swabs using the following procedure: after addition of 250 µl 10 mM Tris-HCl (pH 7.4) the sample was heated at 96 degrees Celsius for 10 minutes. After mixing for 10 seconds the swabs were removed and the sample was centrifuged (14.000 rpm).

### Genotyping

All samples were genotyped for the *TLR2* -16934 T>A (NCBI SNP CLUSTER ID: rs4696480), *TLR2*+2477 G>A (rs5743708), *TLR4*+896 A>G (rs4986790), *NOD1*+32556 (T->GG) (rs6958571), *NOD2*+2209 C>T (rs2066844), *NOD2*+2722 G>C (rs2066845), *NOD2*+3020 ins C (rs5743293), *CASP1*+8404 A>G (rs2282659), and *TRAIL* -692 T>C (rs365238) SNPs by real-time PCR using the TaqMan AbiPrism® 7000 Sequence Detection System (Applied Biosystems, UK) with the standard TaqMan protocol and the the LightCycler® 480 System (Roche Applied Science, US). Results were analyzed by two independent researchers. *TLR9* -1237 T>C (rs5743836) and *TLR9*+2848 G>A (rs352140) were genotyped in a previous study [5] and were used in this multigene study.

### Statistics

Prior to this study power analyses were performed using G*Power 3.1. Since no prior SNP frequencies in BM patients with hearing loss was available we had to estimate the distribution in cases on similar studies and experience.

Within selected severity groups, we compared the distribution of SNP alleles. For statistical analysis, SPSS for Windows 17.0 (IBM Corporation, Somers, New York) and Graphpad Instat Prism® 5 were used. Genotype frequencies of all 11 SNPs were compared between the aforementioned severity groups. Genotypic models of inheritance were used to assess the relation between genetic and clinical variables. Significant associations were further explored for recessive or dominant models. Histograms were used to assess normality. T-tests, Mann-Whitney U tests, and χ^2^ test or Fisher’s exact tests were used where appropriate. Outliers were excluded (<4%) by the Grubbs’ test (*p*<0.01) before continuous testing. Next, continuous variables were dichotomized based on clinical relevant cut-off points as described in literature [44], [45] or as used in clinical practice. After Holm-Bonferroni correction for multiple testing, p-values <0.003 were considered to be statistically significant.

### Multigene Analysis

Combinations of SNPs within the same gene or in the same biological pathway were studied in variables that showed a significant or trend association in one of the SNPs. Studied combinations of SNPs are: *TLR2* -16934, *TLR2*+2477, and *TLR4+896* (stimulating MyD88 via TIRAP and triggering the intracellular signaling cascade), *TLR4+896*, *TLR9-1237* and *TLR9+2848* [48] (activating the MyD88 pathway) and the three *NOD2* SNPs (+2209, +2722 and +3020).

Clinical and genetic variables were modeled using backward stepwise logistic regression.
